# Highly Water-Preserving Zwitterionic Betaine-Incorporated Collagen Sponges With Anti-oxidation and Anti-inflammation for Wound Regeneration

**DOI:** 10.3389/fcell.2020.00491

**Published:** 2020-07-15

**Authors:** Anqi Chen, Ying An, Wen Huang, Tengxiao Xuan, Qianwen Zhang, Mengqi Ye, Sha Luo, Xuan Xuan, Huacheng He, Jie Zheng, Jiang Wu

**Affiliations:** ^1^School of Pharmaceutical Sciences, Key Laboratory of Biotechnology and Pharmaceutical Engineering, Wenzhou Medical University, Wenzhou, China; ^2^College of Chemistry and Materials Engineering, Wenzhou University, Wenzhou, China; ^3^Department of Dermatology, The First Affiliated Hospital of Wenzhou Medical University, Wenzhou, China; ^4^Department of Chemical, Biomolecular, and Corrosion Engineering, The University of Akron, Akron, OH, United States; ^5^Department of Polymer Engineering, The University of Akron, Akron, OH, United States

**Keywords:** wound dressing, zwitterionic betaine, collagen, oxidative stress, water preserving

## Abstract

A core problem in wound healing – with both fundamental and technological significance – concerns the rational design of bioactive and moist microenvironments. Here, we design a new class of zwitterionic betaine-incorporated collagen sponges (BET@COL) with integrated anti-oxidation and anti-inflammatory properties for promoting wound healing in a full-thickness wound model. The presence of zwitterionic betaine in a 3D network structure of collagen enables tightly bound and locked water molecules inside sponges via ionic solvation and confinement effect, while the integration of this amino acid also empowers the sponge with anti-oxidation and anti-inflammatory functions. *In vitro* results demonstrated that BET@COL collagen sponges strongly preserved water content up to 33.78 ± 0.78% at the 80th min at 37°C (only 0.44 ± 0.18% in control), and also exhibited high cell biocompatibility. Further, BET@COL collagen sponges with different betaine contents were applied to a full-thickness cutaneous wound model in mice, followed by a systematical evaluation and comparison of the effect of preserved water on wound healing efficiency *in vivo*. The optimal BET@COL collagen sponges were able to maintain high water content (e.g., moist microenvironment), suppress oxidative stress, improve anti-inflammation, all of which impose synergetic healing effects to promote wound closure, granulation formation, re-epithelization, collagen deposition and angiogenesis. This work demonstrates a new material as a promising candidate for wound dressing.

## Graphical Abstract

Schematic diagram of betaine-incorporated collagen sponge for dermal wound healing with both highly water retentivity and anti-oxidative property.

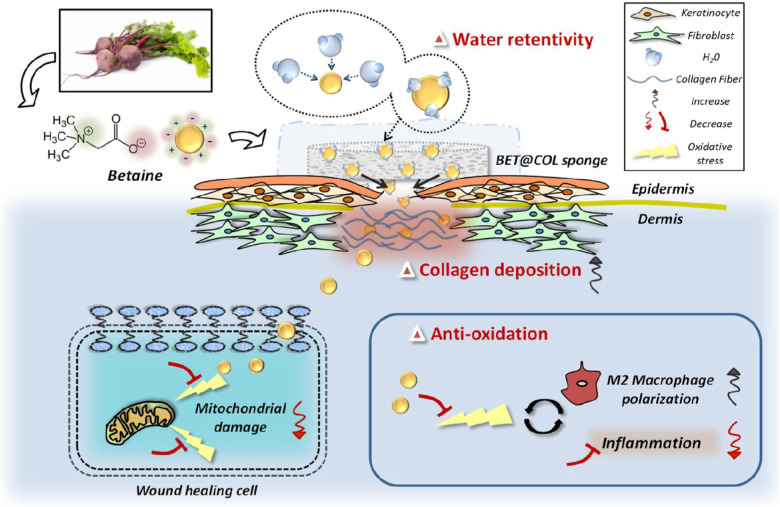

## Introduction

Dermal wound healing is a sophisticated process mainly involving several largely overlapping phases including inflammation, proliferation and remodeling. It is generally accepted that water plays a very important role in maintaining the biofunctions of 90% human soft tissues (not limited to human skin) (Warner et al., 1988; Ousey et al., 2016). Specifically, while human skin contains approximately 30% of water, water content can go up to 70% in the viable epidermis and drop to 15–30% at a junction between the stratum granulosum (SG) and the stratum corneum (SC). Thus, water is not simply and uniformly distributed in human skin, and this water distribution becomes even more complex and dynamic around wound tissues. When a cutaneous wound has just happened, the skin rapidly responds to this external damage by introducing growth factors and proteinases to mediate the healing process, which begin to gather and accumulate fluid exudates (Atiyeh et al., 2002). Afterward, as the healing proceeds, a provisional extracellular matrix (ECM) starts to form as the primary component of wound microenvironment, followed by a long period of remodeling to complete the whole healing process (Warner et al., 1988; Ousey et al., 2016). A moist wound microenvironment was previously demonstrated to facilitate the process of re-epithelialization by benefiting keratinocyte migration through preventing scab formation (Winter, 1962; Hinman and Maibach, 1963). In addition, it provides wounds with more lasting contact with uninfected wound fluid, which favors the proliferation of healing-associated cells (Katz et al., 1991). Additionally, super-hydrated (e.g., only saline solution) environment could accelerate the wound healing process especially at the inflammatory and proliferative phases as compared to dry wounds (Dyson et al., 1988). Conversely, a dry healing environment caused by simple exposure of the wound to the air or by traditional gauze dressing coverage was reported to dehydrate the wound site, cause adhesion and retard the healing process (Cho and Lo, 1998). Therefore, new types of moisture-retentive materials have been developed to create an ideal moist healing environment to efficiently promote wound healing, such as hydrogels (Fonder et al., 2007). Numerous studies have been performed with special focus on the development of water-retentive hydrogels (e.g., gelatin-based hydrogels) for wound dressings (Zhao et al., 2016, 2017; Sun et al., 2017).

However, it is still a challenge to generate water transportation and preservation from simple hydrogels. While hydration promotes wound healing via re-epithelialization, there is still a of clear definition to determine the “optimal” or “balanced” moist environment for wounds, due to the complex nature of skin (Elias and Wakefield, 2011). Therefore, development of novel long-term water preserving dressing materials to efficiently cope with wound exudates and the moist environment, would be a desirable approach for dermal wound application. It was also found that oxidative damage might be one of the most detrimental consequences of dehydration, e.g., oxidation level in yeast cells increased for more than 10-fold after dehydration, indicating that water depletion generates more oxidative stress (Pereira Ede et al., 2003; Franca et al., 2007). These findings suggest a close relationship between water preservation and cellular anti-oxidation during wound repair. Additionally, it has been found that in the wound fluid of mice, along with the inflammation period, oxidative stress occurs with the dramatically increasing production of hydrogen peroxide [H_2_O_2_, a member of the reactive oxygen species (ROS) family] (Roy et al., 2006). It should also be noticed that at the inflammation phase of wound repair, an imbalance between ROS and its detoxification system would occur, leading to a disproportionately high level of oxidative stress in the wound site (Hameedaldeen et al., 2014). This oxidative stress has mutual promoted effects with the inflammatory reaction and plays a critical role in the wound healing process (Lan et al., 2013). In the inflammatory phase, the “respiratory burst” caused by neutrophil infiltration leads to the production of free radicals, which results in mitochondrial and DNA damage, lipid peroxidation, the inactivation of free radical scavenger enzymes and cell apoptosis or necrosis (Babior, 1978; Lennon et al., 1991; Wiseman and Halliwell, 1996; Cadenas and Davies, 2000). Thus, attenuating oxidative stress response turns out to be an effective strategy for the treatment of acute and chronic wounds, and the development of drugs with anti-oxidative stress function, topically applied to dermal wounds, show a promising future (Cao et al., 2018; Zhu et al., 2018). For all these reasons, multifunctional wound dressings have been under intensive investigations. In recent years, a variety of synthetic material-based wound dressings were fabricated with salutary effects including adhesive, antibacterial, and antioxidant properties for improving healing outcome (Li et al., 2019; Liang et al., 2019; He et al., 2020).

Considering both water preserving and anti-oxidative stress properties, the naturally originated compound, zwitterionic betaine, was considered for its special properties. As a small N-trimethylated amino acid, betaine widely exists in plants, animals and microorganisms (Craig, 2004). It is extensively recognized as an organic osmolyte, and in plants serves as a protector against drought, high salinity and osmotic stress (Yancey et al., 1982). Betaine is particularly characterized by its lasting moisture-retentive ability and is widely used in skin care products due to its chemical structure featuring a hydrophilic head which attracts water molecules (Wattanaploy et al., 2017). Moreover, previous studies suggested that betaine helped improve the hydration state of the epithelium (Nicander et al., 2003). It is noteworthy that, except for its water preserving function, in mammals, the anti-oxidative effect of betaine has been revealed by numbers of studies. Systematic administration of betaine was reported to decrease lipid peroxide levels and increase glutathione (GSH) levels, which indicated its anti-oxidative ability (Balkan et al., 2004). Betaine supplementation also alleviated liver fibrosis by inhibiting both oxidant and inflammatory processes (Tsai et al., 2015; Bingul et al., 2016). A wide range of evidence has shown that systematic administration of betaine restrained the oxidative stress of the target organ, while its local application on dermal skin has seldom been studied.

Herein, the anti-oxidation effects and mitochondria protection of betaine against oxidative damage on NIH 3T3 fibroblasts were first investigated. Then, a varied amount of betaine was loaded into collagen sponges (initially obtained from rat tail tendon) to form BET@COL dressing. With the addition of betaine, there was a significant increase in the water-preserving properties of collagen sponges. Then this series of BET@COL with varied betaine loadings was applied to the full-thickness wound model of C57BL/6 mice. Results showed that, the BET@COL (4 mg) group exhibited the best wound enclosure rate, granulation formation and collagen deposition. Therefore, this BET@COL dressing with satisfactory therapeutic effects on dermal wounds regeneration as a hydrogel substitute showed great potential in future practical clinical use for acute and chronic wound care.

## Materials and Methods

### Materials and Reagents

Dulbecco’s modified Eagle’s medium (DMEM), phosphate buffer saline (PBS), penicillin and streptomycin were purchased from Gibco BRL, Invitrogen Corp., (Carlsbad, CA, United States). Fetal bovine serum (FBS) was obtained from Hyclone, Thermo Scientific (United States). Cell counting kit-8 (CCK8) reagent, bovine serum albumin (BSA), DAPI, hematoxylin and eosin dyes, RIPA lysis buffer (P0013B) and Phenylmethanesulfonyl fluoride (PMSF, ST506) were purchased from Beyotime^®^ Biotechnology (China). Betaine and hydrogen peroxide (H_2_O_2_) were obtained from Sigma-Aldrich LLC (United States). Triton X-100 and Masson’s trichrome staining kit were purchased from Solarbio Science & Technology Co., Ltd (China). Primary rabbit monoclonal to heme oxygenase-1 (HO-1, ab68477), rabbit polyclonal antibody to cytokeratin (ab9377), mouse polyclonal antibody to CD68 (ab955), rabbit polyclonal antibody to CD163 (ab182422), donkey anti-rabbit IgG Alexa Fluor^®^ 647-conjugated secondary antibody (ab150075), donkey anti-rabbit IgG Alexa Fluor^®^ 488-conjugated secondary antibody (ab150073) and donkey anti-mouse IgG Alexa Fluor^®^ 647-conjugated secondary antibody (ab150111) were obtained from Abcam (United Kingdom). Goat anti-rabbit (H + L) HRP secondary antibody was obtained from Bioworld Technology (United States). FITC labeled Phalloidin (40735ES75) and JC-1 mitochondrial membrane potential assay kit (40706ES60) were obtained from Yeasen Biotech. Co., Limited (China). Goat anti-rabbit horseradish peroxidase-conjugated secondary antibody was purchased from Pierce Biotechnology (United States). Bicinchoninic acid (BCA) reagent was obtained from Thermo (United States). Polyvinylidene fluoride (PVDF) membrane and fat free milk were purchased from Bio-Rad (United States). Tween 20 was obtained from Aladdin Chemistry Co., Ltd (China).

### Cell Lines and Cell Cultures

A mouse fibroblast cell line NIH 3T3 was purchased from American Type Culture Collection and cultivated in DMEM containing 10% FBS, 100 unit/mL penicillin and 100 μg/mL streptomycin in a controlled incubator at 37°C with an atmosphere of 5% CO_2_.

### Cell Viability Assays

To measure the effects of betaine on cell viability, NIH 3T3 fibroblast cells were seeded at 8.0 × 10 (Atiyeh et al., 2002)cells per well in 96-well plates and cultured in serum containing DMEM mentioned above for 24 h. Then the origin medium was replaced by fresh medium (100 μL/well) with the addition of betaine at various concentration of 0, 2, 5, 10, 50, and 100 mM, respectively. After 12 h treatment and wash with PBS, fresh medium (100 μL/well) containing 10% CCK8 reagent was added to each well for another incubation of 2 h at 37°C. The cell viability was determined by measuring the absorbance at 450 nm using a microplate reader (Molecular Devices, SoftMax^®^ Pro 5, United States).

To measure the protective effects of betaine on cells against oxidative damage, NIH 3T3 fibroblasts cells were seeded and cultivated for 24 h as described above. Then the origin medium was replaced by fresh medium (100 μL/well) with the addition of betaine at the concentration of 0, 2, and 5 mM, respectively. After betaine pretreatment at 37°C for 5 h, the medium was then replaced with fresh medium (100 μL/well) with the addition of 50 μM H_2_O_2_ for another incubation of 4 h at 37°C. At the end of incubation, each well was washed with PBS and fresh medium (100 μL/well) containing 10% CCK8 reagent was added for another incubation of 2 h at 37°C. Finally, the absorbance was determined at 450 nm.

### Cell Immunofluorescence

Thr NIH 3T3 Fibroblast cells were seeded at 2.0 × 10 (Ousey et al., 2016)cells per well in 6-well plates and cultured for 24 h followed by the replacement of medium by fresh medium (4 mL/well) with 0 and 2 mM betaine, respectively. After betaine pretreatment at 37°C for 5 h, the medium was replaced with fresh medium (4 mL/well) with 50 μM H_2_O_2_ for another incubation of 4 h at 37°C. The medium was then carefully removed and washed 3 times with PBS. Cells were fixed with 4% paraformaldehyde for 30 min at 4°C, and incubated in 5% BSA containing 0.1% Triton X-100 for 40 min at 37°C. After that, cells were incubated with a rabbit monoclonal antibody to HO-1 (1:200) diluted in PBS containing 1% BSA overnight at 4°C. Cells were then thoroughly washed with PBS and incubated with a donkey anti-rabbit IgG Alexa Fluor^®^ 647-conjugated secondary antibody (1:1000) and FITC labeled Phalloidin (1:100) diluted in PBS at 37°C for 1 h in the dark. Finally, cells were stained with DAPI to visualize the nuclei. Fluorescence images were captured using a Nikon confocal laser microscope (Nikon, A1 PLUS, Tokyo, Japan). Image-Pro Plus 6.0 software was used to count positive fluorescent samples in each fluorescent image, followed by further statistical analysis.

### Western Blotting

The total cell lysates of NIH 3T3 fibroblasts after different treatments were collected with RIPA lysis buffer (with 1% PMSF) for 15 min on ice. After centrifugation (12,000 × *g*, 10 min, 4°C), the supernatant was collected and the protein concentration of which was then quantified using BCA reagents. After that, equal amounts of protein samples from each group were separated through a Bis-Tris polyacrylamide gel (12%) under 80 V and transferred to a PVDF blotting membrane, which was then blocked with 5% skimmed milk in TBST (10 mM Tris–HCl, 100 mM NaCl and 0.1% Tween 20) for 1.5 h at room temperature on a rotary shaker and then incubated with a rabbit monoclonal antibody to HO-1 (1:1000) diluted in TBST overnight at 4°C. The membrane was washed with TBST three times and incubated with a goat anti-rabbit horseradish peroxidase-conjugated secondary antibody (1:8000) for 2 h at room temperature on a rotary shaker. The protein bands on the PVDF membrane were visualized using ChemiDicTM XRS + Imaging System (Bio-Rad), and the signal intensities of which were quantified using ImageJ software. Band intensities were normalized to GAPDH.

### Mitochondrial Membrane Potential Examination

The JC-1 mitochondrial membrane potential assay kit was used to test mitochondrial membrane potential △Ψm. Briefly, NIH 3T3 Fibroblast cells were pretreated with 2 mM betaine for 3 h, followed by a 100 μM H_2_O_2_ stimulation for 1 h. 20 min before the termination of stimulation, the cells of positive control group were incubated with 2 mL medium containing 2 μL carbonyl cyanide 3-chlorophenylhydrazone (CCCP, 10 mM) which induces cell apoptosis. Then cells were washed with PBS 3 times and cell suspensions were obtained after trypsinization. 5,5′,6,6′-Tetrachloro-1,1′,3,3′-tetraethylbenzimidazolylcarbocyanine iodide (JC-1) solution from JC-1 mitochondrial membrane potential assay kit was then used to treat cells for 20 min at 37°C. Cells were then washed with JC-1 staining buffer solution for three times. After that, cells were transferred to a black 96-well plate (100 μL per well) and fluorescence of the mitochondria monomers (excitation wavelength: 485 nm; emission wavelength: 535 nm) and aggregates (excitation wavelength: 550 nm; emission wavelength: 600 nm) were determined using a microplate reader (Molecular Devices, SoftMax^®^ Pro 5, United States). The △Ψm status of mitochondria was presented by the ratio of red to green fluorescence. For mitochondria observation, cells after treatment were incubated with JC-1 solution for 20 min at 37°C, washed with JC-1 staining buffer solution for three times, fixed with 4% paraformaldehyde for 30 min at 4°C and finally stained with DAPI to visualize the nuclei. Fluorescence images were captured using a Nikon confocal laser microscope (Nikon, A1 PLUS, Tokyo, Japan).

### Collagen Sponge Preparation

The type I collagen was extracted from the tails of SD rats provided by the Laboratory Animals Center of Wenzhou Medical University. First, rat tails were rinsed and sterilized with 75% ethanol. Then the tendons in the tails were carefully extracted, cut into small pieces and placed at 4°C for 48 h. After tendons became dry, they were dissolved in acetic acid (0.1 M) for 5 days. After that, the suspension was centrifuged and put in tissue culture dishes with its pH adjusted to 7.0. Then it was frozen at −80°C and freeze-dried to form collagen sponges. The collagen sponges were punched into round pieces with a diameter of 7 mm and then disinfected with ultraviolet radiation for *in vivo* application.

The inner spongy structure of collagen sponges was observed using a scanning electron microscopy (SEM, VEGA3 TESCAN). Firstly, the collagen sponges were dried in liquid nitrogen. Secondly, they were gold sputtered for 60 s under high-vacuum conditions by a Desk II gold sputter coater (Denton Vacuum, Morristown, NJ, United states). The inner structure was finally observed and captured.

### Water Preservation Examination

40 μL 0.9% saline or 0.2 g/mL betaine solution was added to round collagen sponges 7 mm in diameter. After absorption, each piece of collagen sponge was immediately weighed (recorded as W_0_) and placed in an incubator chamber at 37°C. At each determined time point (the Nth minute), each collagen sponge containing a different solution was weighed (recorded as W_*N*_) and recorded for further analysis of water preservation. The weight of non-evaporable solute (betaine or sodium chloride) was recorded as W_*S*_. Water preservation was calculated as following:

Water Preservation (%) = (W_*N*_-W_*S*_)/(W_0_-W_*S*_) × 100%

### *In vitro* Betaine Release Profile

First, BET@COL with different drug loads (2, 4, and 8 mg) were immersed in 400 μL saline, respectively. At each determined time point (0.5, 1, 2, 3, 4, 5, and 6 days) the saline of each group was collected for further concentration detection and fresh saline was added. All the samples were stored at 4°C before detection. Then, we prepared betaine solutions at gradient concentrations of 0.3, 0.6, 1.2, 1.5, 1.8, 2.4, 3.0, 4.2, and 5.4 mg/mL as standards. The betaine amount was detected by high performance liquid chromatography (HPLC) using an HPLC System (Agilent 1100, United States) with the mobile phase containing CH_3_CN (solvent A) and H_2_O (solvent B) (85:15, v/v) at a flow rate of 0.7 mL/min at 30°C on a Merck Purospher@ STAR RP-18 endcapped (5 μm) Hibar@ RT 250-4.6 HPLC column (Darmstadt, Germany). The wavelength of the evaluation was 195 nm. Three independent samples were tested in each group (*n* = 3). After the detection of standards, a calibration curve was drawn, by which the betaine amounts of the samples were calculated.

### *In vivo* Wound Healing Study

In this study, we used male C57BL/6 mice (8–10 weeks, obtained from the Laboratory Animals Center of Wenzhou Medical University). All experiments were performed in accordance with international ethical guidelines and the National Institutes of Health Guide concerning the Care and Use of Laboratory Animals. Mice were individually anesthetized via intraperitoneal injection with 4% chloral hydrate and the dorsal skin was shaved and sterilized with ethanol. After stitching silicone rings with an internal diameter of 8 mm and thickness of 0.5 mm on the dorsal skin to prevent skin contraction, two round full-thickness wounds with a diameter of 6 mm were created by a biopsy punch (Acuderm^®^ Inc., Fort Lauderdale, FL, United States) per mouse. Mice were randomly divided into five groups: group 1 was applied with 0.9% saline (the control group), group 2 with free betaine solution (4 mg), group 3–5 with BET@COL with different drug loads (2, 4, and 8 mg), similar to the dose-administration used in the previous report (Mahibalan et al., 2016). After application, wounds were covered with a sheet of 3M Tegaderm Film (3M Health Care, Germany) and medical bandages. Dressings were changed per week. We photographed the wounds on day 7, 10, 14, 17, and 20 post surgery and measured the wounds areas using Image-Pro plus. On day 7 and 20, mice were sacrificed after anesthesia and the full-thickness wound tissues were harvested, fixed in 4% paraformaldehyde, embedded in paraffin and sectioned at a thickness of 5 μm using a microtome (LEICA RM2235, Germany) for further investigation.

### Histopathological Examination

Hematoxylin and eosin (H&E) staining and Masson’s trichrome staining were performed here. First, samples were dewaxed in xylene for 30 min and rehydrated using gradient ethanol. Second, for H&E staining, samples were submerged for 5 min in hematoxylin, 3 min in PBS and then stained with eosin for 2 min. For Masson’s trichrome staining, samples were stained with A1:A2 (1:1) for cell nuclei visualization for 5 min and submerged for 3 s in acid alcohol for differentiation, 5 min in ponceau acid fuchsin solution to stain fibrous tissue, 1 min in 2% acetic acid solution, 30 s in phosphomolybdic acid solution for differentiation, 20 s in aniline blue and 5 min in distilled water. Finally, after dehydration of gradient ethanol and 15 min in xylene, slides were mounted with neutral resin. The stained sections were photographed using a Nikon microscope (Nikon, Tokyo, Japan).

### Immunohistochemistry and Immunofluorescence

For immunohistochemical staining, after deparaffinization and rehydration, the sections were incubated in 3% H_2_O_2_ for inactivation of the endogenous peroxidase for 15 min and in 5% BSA for blockage of the non-specific binding sites at 37°C for 30 min. For immunohistochemical staining, the sections were incubated with a rabbit polyclonal antibody to cytokeratin (1:300) diluted in PBS containing 1% BSA at 4°C overnight. After being thoroughly washed with PBS, the sections were incubated with a goat anti-rabbit (H + L) HRP secondary antibody (1:1000) diluted in PBS at 37°C for 60 min, then counterstained with hematoxylin, mounted with neutral resin and finally photographed using a Nikon microscope (Nikon, Tokyo, Japan). For immunofluorescence staining, after deparaffinization and rehydration, the sections were incubated in 5% BSA for blockage of the non-specific binding sites at 37°C for 30 min, followed by incubation with a rabbit monoclonal antibody to HO-1 (1:200), a mouse polyclonal antibody to CD68 (1:200) and a rabbit polyclonal antibody to CD163 (1:200) diluted in PBS containing 1% BSA at 4°C overnight. After being thoroughly washed with PBS, the sections were incubated with a donkey anti-rabbit IgG Alexa Fluor^®^ 488-conjugated secondary antibody (1:1000), a donkey anti-mouse IgG Alexa Fluor^®^ 647-conjugated secondary antibody (1:1000), and a donkey anti-rabbit IgG Alexa Fluor^®^ 647-conjugated secondary antibody (1:1000) diluted in PBS at 37°C for 60 min in the dark, according to the primary antibodies. The sections were then stained with DAPI, mounted with antifade mounting medium and photographed using a Nikon confocal laser microscope (Nikon, A1 PLUS, Tokyo, Japan). Image-Pro Plus 6.0 software (Nikon, Tokyo, Japan) was used to count positive fluorescent samples in each fluorescent image, followed by further statistical analysis.

### Statistical Analysis

All data were expressed as mean ± standard error (SE). Statistical differences were performed using one-way analysis of variance (ANOVA) followed by Tukey’s test with GraphPad Prism 5 software (GraphPad Software Inc., La Jolla, CA, United States). For all tests, ^∗^*p*-value < 0.05, ^∗∗^*p*-value < 0.01, ^∗∗∗^*p*-value < 0.001.

## Results and Discussion

### *In vitro* Effects of Betaine on NIH 3T3 Fibroblasts

**FIGURE 1:**
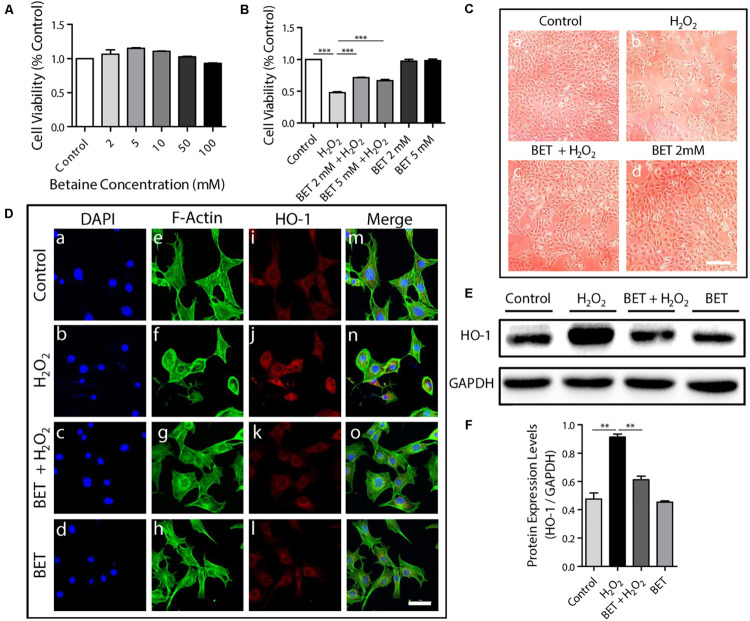
The cyto-compatibility and anti-oxidative stress property of betaine *in vitro*. **(A)** NIH 3T3 cell viability after 12 h’ betaine treatment of different concentration, *n* = 6. **(B)** NIH 3T3 cell viability after 4 h’ H_2_O_2_ (50 μM) exposure with or without pretreatment of 5 h’ betaine (2 mM and 5 mM), *n* = 6. **(C)** NIH 3T3 cell morphology observed by optical telescope: **(a)** control **(b)** after 4 h’ H_2_O_2_ (50 μM) exposure **(c)** after 4 h’ H_2_O_2_ (50 μM) exposure with pretreatment of 5 h’ betaine (2 mM). Scale bar: 200 μm. **(d)** after 5 h’ betaine treatment. **(D)** HO-1 immunofluorescence staining of NIH 3T3 cells after 4 h’ H_2_O_2_ (50 μM) exposure with or without pretreatment of 5 h’ betaine (2 mM). The figures share the same scale bar of 50 μm. **(E,F)** HO-1 expression analysis by Western blot, *n* = 3. NIH 3T3 cells were treated as described in **(D)**. BET: betaine. Statistical differences were performed using ANOVA. ***p < 0.001, **p < 0.01.

To affirm the cyto-compatibility of betaine on fibroblasts, one of the main cell types of the skin which also plays an indispensable part during the wound process (Takeo et al., 2015), NIH 3T3 fibroblasts were treated with betaine at various concentrations of 0, 2, 5, 10, 50, and 100 mM. The consequent graph of cell viability measured by CCK8 assay shown in Figure 1A suggested that betaine exerted no toxic effect to NIH 3T3 fibroblasts. At the relatively low concentrations, betaine exhibited slight effects of promoting cell proliferation. To mimic the oxidative stress damage which occurs at the wound site, we conducted H_2_O_2_-induced oxidative damage assays on NIH 3T3 fibroblasts *in vitro.* For exploring the protective effects of betaine against oxidative stress, NIH 3T3 fibroblasts were pretreated with 0, 2, and 5 mM betaine in medium for 5 h, followed by the addition of 50 mM H_2_O_2_ for 4 h. We should note that cell biocompatibility was tested under normal physiological condition, while cell protection tests against oxidative stress were performed by using H_2_O_2_ to treat cells. Additionally, upon H_2_O_2_ stimulation, even the low-dose betaine groups (2 mM and 5 mM) both showed significant protective effects, indicating the excellent cell protective effect under oxidative stress of betaine. While the 5 mM group showed the slightly lower cell viability than the 2 mM group, no statistical significance was observed between the two groups (*p* = 0.0611). As seen in Figure 1B, H_2_O_2_ stimulation caused an obvious cell death with only 47.7 ± 2.6% living cells compared with the untreated cell, which was significantly rescued by the pretreatment of 2 mM betaine (71.3 ± 1.2%) and 5 mM betaine (66.6% ± 4.0%), respectively. The photographs in Figure 1C visually displayed the cell morphology and density variation under different treatment, which was in accordance with the result in Figure 1B, further indicating that betaine served as an effective protector of cells resisting oxidative stress *in vitro*.

Heme oxygenase-1 (HO-1) expression was reported to be induced by stimuli including oxidative stress and thus was considered as the indicator of oxidative stress levels (Schipper et al., 2006). To further verify betaine’s effects upon oxidative stress, HO-1 was used as an intracellular oxidative stress levels detector. Immunofluorescence staining of HO-1, counterstained with F-actin (stained by FITC-labeled Phalloidin) for cytoskeleton of NIH 3T3 fibroblasts was shown in Figure 1D. The H_2_O_2_ group exhibited obviously higher fluorescence intensity of HO-1 in contrast with the control group with cell shrinkage (Figures 1D,b). However, with the pretreatment of 2 mM betaine, HO-1 expression was reduced compared with the H_2_O_2_ group whereas the cell morphologies were much closer to the untreated group (Figures 1D,c), indicating a good antioxidant effect of betaine on NIH 3T3 cells. Then HO-1 protein expression was determined by western blotting as shown in Figures 1E,F. The 4 h exposure to H_2_O_2_ caused a significant increase of HO-1 expression in NIH 3T3 fibroblasts by 1.92-fold (*p* < 0.01) compared with control cells. Cells pretreated with 2 mM betaine exhibited a slighter increase of HO-1 expression when exposed to H_2_O_2_ by 1.29-fold. Thus, betaine pretreatment contributed to a decrease of HO-1 expression in cells exposed to H_2_O_2_, suggesting that the betaine had protected the cells from oxidative damage by lowering the oxidative stress level of cells suffering oxidative damage, which also can explain the increased cell viability in Figure 1B.

**FIGURE 2:**
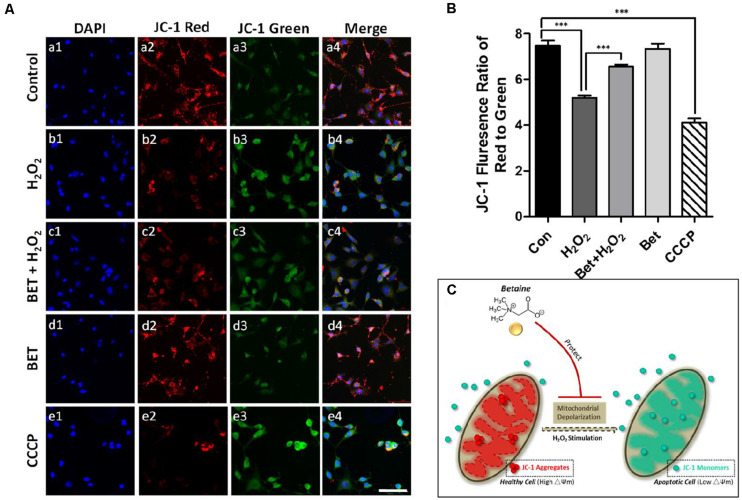
Betaine protected the mitochondria in NIH 3T3 cells against oxidative damage. **(A)** Fluorescence staining of JC-1 for mitochondria membrane potential. Red: mitochondrial aggregates. Green: mitochondrial monomers. Scale bar = 100 μm. **(B)** The ratio of red to green fluorescence, *n* = 3. CCCP: positive control. BET: betaine. **(C)** Schematic showing the mitochondrial protective effect of BET against H_2_O_2_ damage. Statistical differences were performed using ANOVA, ***p < 0.001.

Oxidative stress response has significant effects on mitochondria, which influences mitochondrial membrane potential, leading to mitochondrial depolarization (Lieven et al., 2003). During the wound healing process, the inevitable elevated oxidative stress reaction, along with inflammation, induces cell apoptosis, during which mitochondria plays an important role (Granville et al., 2001). In order to investigate mitochondrial status change in wound healing-associated cells under oxidative stress stimulus, we performed examinations of mitochondrial membrane potential (△Ψm) using JC-1 staining. In healthy cells, mitochondria have a high △Ψm, shown as red stained aggregates formed by JC-1 dye. While in cells with mitochondria damage, △Ψm is lower and JC-1 will exhibit green fluorescence as monomers. As observed in Figures 2A,a1–a4, NIH 3T3 fibroblasts in the control group had a high intensity of red fluorescence with a relatively low level of green fluorescence, indicating a high △Ψm of mitochondrion. After H_2_O_2_ (100 μM) exposure for 1h, a significant reduction in red fluorescence could be seen. Meanwhile, the loss of △Ψm caused a strong green fluorescence (b1-b4), whereas the pretreatment with betaine attenuated △Ψm loss resulted from H_2_O_2_ stimulus, indicated by a brighter red fluorescence (c1-c4). Additionally, free betaine treatment exerted minimal effects on △Ψm in fibroblasts (d1-d4). CCCP (carbonyl cyanide m-chlorophenylhydrazone) is a protonophore uncoupling agent for oxidative phosphorylation and a potent inducer for increasing membrane proton conductance of △Ψm (Kasianowicz et al., 1984; Livingston et al., 2019; Soutar et al., 2019), thus CCCP is served as a positive control for comparison (e1-e5). A more accurate measure of △Ψm change after different treatments was performed by calculating the ratio of red to green fluorescence, the low level of which represented the loss of △Ψm and a damaged mitochondria condition. In Figure 2B, in assistance with images in Figure 2A, the exposure to H_2_O_2_ led to a dramatic decrease of fluorescence ratio (5.21 ± 0.10) compared to the control cells (7.48 ± 0.23), which was measured using a microplate reader. While betaine pretreatment significantly rescued the loss of △Ψm with an elevated ratio of 6.56 ± 0.08 (*p* < 0.001). The mitochondrial protective effect of BET against H_2_O_2_ damage was presented in the diagram in Figure 2C.

### Characterization of BET@COL

**FIGURE 3:**
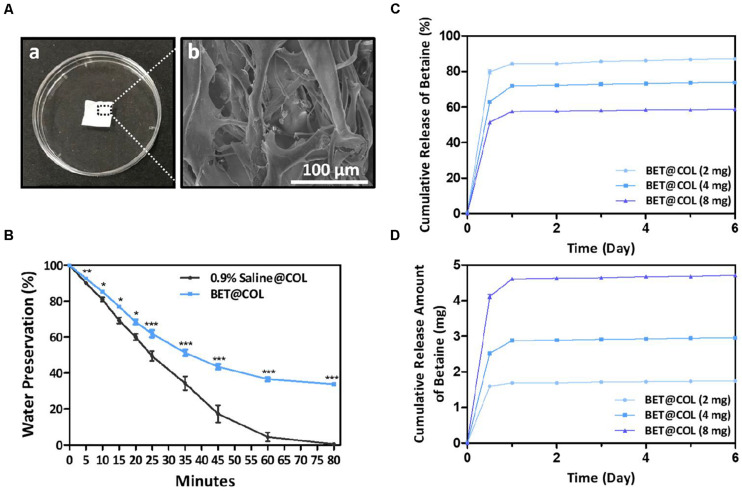
Characteristics and betaine release of BET@COL. **(A)** Photographs of thin collagen sponge dressing and its inner instruction **(a)** of collagen sponge dressings by SEM **(b)**. Scale bar: 100 μm. **(B)** Water preservation percentage of BET@COL, *n* = 3. **(C,D)** *In vitro* release profile of BET@COL. The cumulative release percentage **(C)** and the cumulative release amount **(D)** of betaine of different drug load (2 mg, 4 mg, and 8 mg) measured by HPLC, *n* = 3. BET, betaine; COL, collagen. Statistical differences were performed using ANOVA. ***p < 0.001, **p < 0.01, *p < 0.05.

In order to provide a supportive moisturizing scaffold for wound regeneration, we encapsulated betaine into a thin collagen material derived from rat tail collagen (Figures 3A,a). The SEM results (Figures 3A,b) showed an inner loose spongy construction of collagen, which was speculated to have good absorbency to achieve better drug load and the wound exudation absorption as well. We next investigated the water preserving ability of betaine in collagen sponges by comparing to that of 0.9% saline-containing collagen sponges. An equivalent volume of betaine solution and saline was evenly added to the collagen sponge for a complete absorption, followed by dehydration at 37°C. Weight of the collagen sponges of two groups were obtained at different time points and the water preservation (%) was calculated and shown in Figure 3B. At each time point, the betaine group showed significantly higher water preservation percentage compared with the saline group, indicating an excellent water preserving function of betaine. On the 80th min, the saline containing collagen sponge had only 0.44 ± 0.18% water preservation, while the betaine group remained 33.78 ± 0.78% (*p* < 0.001). Thus the addition of betaine endowed the collagen sponge with significantly improved water retentivity, which could provide a much more moist environment on the wound surface in favor of epithelial crawling.

Next, after absorbing betaine solutions containing various drug amounts of 2, 4, and 8 mg, BET@COL were investigated for betaine release rate by HPLC at determined time points. The calibration curve for betaine was found to have good linearity over the range of 0.6–5.4 mg/mL (*r* = 0.997). Figure 3C showed the cumulative release percentage of betaine. All the groups reached the release peak on day 1, with the maximum cumulative release of 87.2 ± 0.1% (2 mg), 73.9 ± 0.1% (4 mg) and 59.0 ± 0.1% (8 mg) during the whole releasing process. The cumulative release amounts of betaine were 1.7 ± 0.003 mg (2 mg), 3.0 ± 0.004 mg (4 mg) and 4.7 ± 0.01 mg (8 mg) presented in Figure 3D. In our acute wound model experiments, the “burst release” of betaine in the early phase helps to alleviate the inflammatory response through its anti-oxidative stress effect. Additionally, this *in vitro* release is likely different from the release profile *in vivo* at wound sites. It is expected that during the wound healing process, the collagen sponge would be further hydrolyzed by wound collagenases (Chattopadhyay and Raines, 2014), so as to gradually release the rest of the betaine entrapped in the sponge for the purpose of maintaining the gradual healing. According to Figures 3C,D, as the incorporated betaine increased, the cumulative release percentage of betaine decreased, but the cumulative release amounts of betaine increased. This could be due to the strong intermolecular electrostatic interactions between zwitterionic betaine pairs and between betaine and collagen matrix, which could tighten the sponge networks and thus impose an additional barrier for releasing betaine from the sponge (Zheng et al., 2018; McCoy et al., 2019).

### The Wound Healing Promoting Effects of BET@COL

**FIGURE 4:**
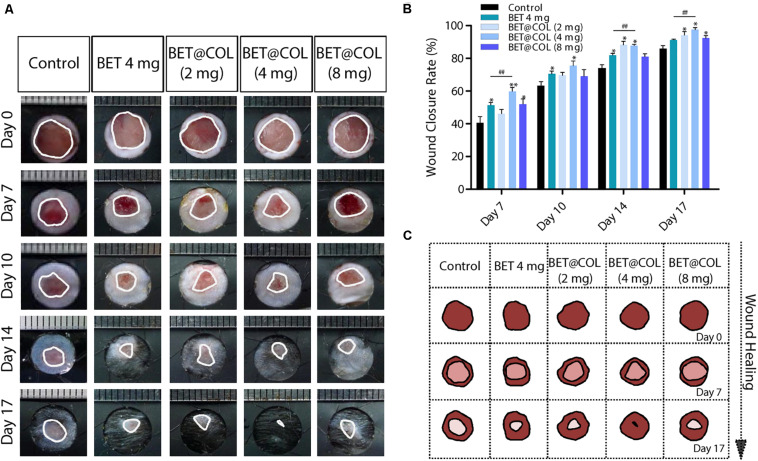
BET@COL significantly resurfaced dermal full-thickness wound. **(A)** Representative pictures of the macroscopic wound healing processes of the control, free betaine solution treated and BET@COL treated wounds. The rulers in the pictures are shown in millimeters. **(B)** Statistical wound closure rates of the groups mentioned above, *n* > 6. **(C)** Schematic diagram of the traces of *in vivo* wound closure over 17 days. BET, betaine; COL, collagen. Statistical differences were performed using ANOVA. **p < 0.01, *p < 0.05, compared to control group, ##*p* < 0.01.

Since the protective effects against oxidative damage and the water retentivity of betaine *in vitro* were confirmed as mentioned above, we performed animal experiments to investigate its effects on the dermal wound healing process. Representative photographs of differently treated wounds at determined time points were shown in Figure 4A. Overall, all BET@COL groups with different drug loads exhibited accelerated wound closure, among which the BET@COL (4 mg) group showed the fastest wound closure rate, suggesting 4 mg as an appropriate drug amount loaded in collagen sponges to achieve better wound healing (Figure 4B). Especially on day seven post-surgery, the healing rate of the control group was 40.73 ± 3.64%, while the BET@COL (4 mg) showed a significantly accelerated rate of 59.92 ± 2.33% (*p* < 0.01). At the end of the observation (day 17), the BET@COL (4 mg) group also had the highest wound enclosure rate of 97.56 ± 1.25% (*p* < 0.01), which was shown as an almost complete wound resurfacing in Figure 4A. In order to make a comparison with the BET@COL (4 mg) group, free betaine solution (4 mg) was also applied to the wounds. Comparing the BET@COL (4 mg) group with the free betaine (4 mg) group, on day 7, 14, and 17 post wounding, the BET@COL (4 mg) group showed a significantly accelerated healing rate (*p* < 0.01), indicating an additional pro-healing function of the collagen. Additionally, the free betaine solution (4 mg) treated group significantly promoted wound closure on day 7, 10, and 14 in contrast with the untreated group. The BET@COL (2 mg and 8 mg) groups exhibited slight healing promoting effects with no general statistical significance. The overall wound healing trends during the observation period in the five groups were illustrated in the schematic diagram in Figure 4C.

**FIGURE 5:**
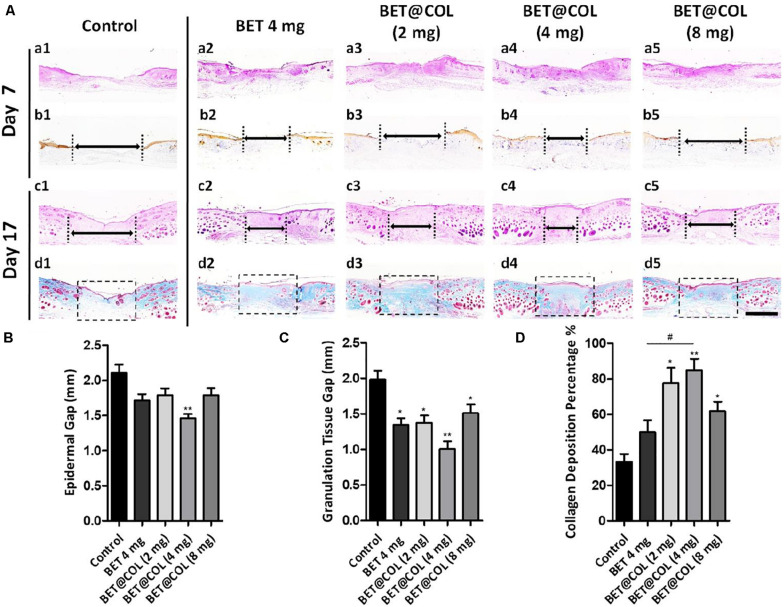
Histological analysis of wound sections on days seven and 17 post treatment. **(A) (a1–a5, c1-c5)** Representative pictures of H&E (Hematoxylin and Eosin) staining of the control, free betaine solution treated and the BET@COL treated wound tissue sections on day seven and day 17. **(b1–b5)** Immunohistochemical staining of cytokeratin of the groups mentioned above on day seven. **(d1–d5)** Masson’s trichrome staining of the groups mentioned above on day 17. Double-headed arrows indicated an epidermal gap **(b1–b5)** or granulation tissue gap **(c1–c5)**. Dotted boxes indicated collagen deposition sites **(d1–d5)**. Scale bar is 1 mm. **(B)** Length of epidermal gap on day seven, *n* > 3. **(C)** Granulation tissue gap on day 17, *n* > 3. **(D)** Collagen deposition percentage on day 17, *n* > 3. BET, betaine; COL, collagen. Statistical differences were performed using ANOVA. **p < 0.01, *p < 0.05, compared to control group, #*p* < 0.05.

The histological constructions of the dermal wounds undergoing different treatment were observed by histological staining on wound tissue sections (Figure 5). The re-epithelialization of the wounds on day 7 was observed by the immunohistochemical staining of cytokeratin, a marker indicating epidermis and hair follicles (Figures 5A,b1–b5). The BET@COL (4 mg) group exhibited the shortest epidermal gap (1.46 ± 0.06 mm) compared with 2.11 ± 0.16 mm of the control group (*p* < 0.01). The wounds treated with free betaine solution (4 mg) also showed an accelerated epidermal regeneration, with an epidermal gap of 1.71 ± 0.09 mm remaining (Figure 5B). The above observations of epidermis crawling were approximately consistent with the macroscopic wound enclosure rate in Figure 4, which may be resulting from the moist wound environment created by BET@COL together with the therapeutic effects of betaine. Granulation tissue is the newly formed construction in the wound bed which is mainly composed of connective tissues and blood capillaries and finally remodeled into mature skin construction (Ali et al., 2016). The granulation tissue gaps on day 17 were measured to evaluate the deep tissue regeneration and the statistical result was shown in Figure 5C. The control group remained an unhealed gap of 1.98 ± 0.12 mm, while the BET@COL (4 mg) group accelerated granulation tissue development from both wound edges, leaving a narrow gap of only 1.01 ± 0.11 mm (*p* < 0.001). Other BET@COL treated groups also showed significant reduction, which was 1.37 ± 0.10 mm for the BET@COL (2 mg) group (*p* < 0.01) and 1.51 ± 0.12 mm for the BET@COL (8 mg) group (*p* < 0.05). For the free betaine (4 mg) group, significantly shorten granulation gap was also observed (1.34 ± 0.10 mm, *p* < 0.01), which was wider than the BET@COL (4 mg) group. Furthermore, the effects of BET@COL on collagen deposition on day 17 were analyzed by Masson’s trichrome staining, by which collagen and nuclei were marked as blue and muscle marked as red (Figures 5A,d1–d5). It was observed that the wounds treated with BET@COL produced obviously more and denser collagen fibers, and the statistical result confirmed that the BET@COL (4 mg) group had a significantly higher collagen deposition percentage of 84.87 ± 6.33% compared with the control group (33.33 ± 4.26%, *p* < 0.01) and the free betaine (4 mg) group (49.95 ± 6.80%, *p* < 0.05). Moreover, the free betaine solution treatment showed no significant difference in collagen deposition compared with the control group, while there appeared a considerable increase in the BET@COL (4 mg) group by 2.55-fold compared with the control group, and the BET@COL groups with a drug loading of 2 mg and 8 mg also showed significantly enhanced collagen deposition in wounds. Furthermore, we used the immunohistochemical staining of α-SMA (α-smooth muscle actin) (Yoshizawa et al., 2015; Seo et al., 2018), a marker for smooth muscle cell and mature vascularization, to evaluate angiogenesis and vascular maturation on 7-day tissue sections (Supplementary Figure S1). As shown by red arrows in Supplementary Figure S1, the wounds treated by BET@COL dressings exhibited a higher density of newly formed blood vessels and larger lumen sizes than both the control and BET (4 mg) groups. Among BET@COL dressings, the BET@COL (4 mg) dressing showed the best angiogenesis, as evidenced by the highest density of blood vessels and the largest vessel size (Supplementary Figures S1B,C). The angiogenesis promoting effect of BET@COL sponges was attributed to a synergistic combination of water-preserved, anti-oxidation, and anti-inflammatory property of zwitterionic betaine in collagen sponge. Specifically, (i) the moister environment created by the BET@COL sponges contributes to the accelerated dermal/wound bed repairs, including epithelialization, cell proliferation, ECM synthesis, and angiogenesis (Rousselle et al., 2019); (ii) a moister environment also prolongs the retention of bFGF and VEGF at wound sites to promote neovascularization; (iii) betaine can effectively suppress the inflammatory responses by minimizing oxidative stress at wound sites, thus boosting the subsequent collagen deposition and angiogenesis (Eming et al., 2009). In conclusion, with the beneficial effects exerted by betaine, in addition with the support of collagen, BET@COL accelerated wound regeneration by prompting re-epithelialization, granulation formation, collagen deposition and angiogenesis.

### BET@COL Restrained Oxidative Stress and Inflammatory Response Upon Wound Healing Process

**FIGURE 6:**
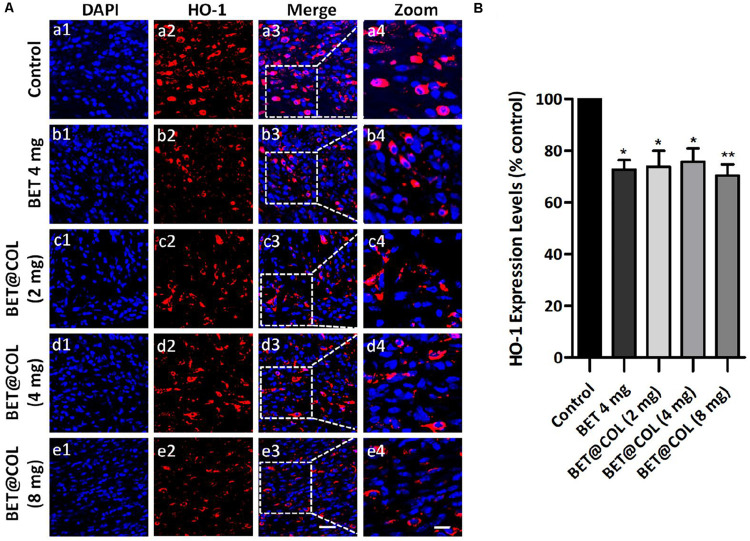
Betaine ameliorates oxidative stress level at wound sites. **(A)** Immunofluorescence photomicrographs of heme oxygenase-1 (HO-1) in wounds for the control group, the free betaine solution (4 mg) group and BET@COL (2 mg, 4 mg, and 8 mg) treated group on day seven. Scale bars: 20 μm [10 μm in magnified images **(a4–e4)**]. **(B)** Quantitative result of immunofluorescence, *n* = 5. BET, betaine; COL, collagen. Statistical differences were performed using ANOVA. **p < 0.01, *p < 0.05, compared to control group.

The *in vitro* anti-oxidative stress and mitochondria-protecting effects of betaine were confirmed on NIH 3T3 fibroblasts, and we wondered if the application of betaine on dermal wounds had such protective effects against oxidative damage caused by skin injuries. We performed immunofluorescence staining of HO-1, an indicator of oxidative stress levels described above, on wound tissue sections on day seven (Figure 6A). The control group exhibited a relatively higher intensity of HO-1 expressing cells in wound center, indicating a stronger oxidative stress response, which was significantly reduced to lower levels after the administration of betaine. Compared with the control group, the free betaine solution (4 mg) treated wounds exhibited 72.65 ± 3.64% (*p* < 0.05) and the BET@COL groups showed decreased levels of 73.80 ± 6.16% (*p* < 0.05), 75.66 ± 5.12% (*p* < 0.05) and 70.44 ± 4.24% (*p* < 0.01) for drug loadings of 2, 4, and 8 mg, respectively (Figure 6B). Thus, we speculated that betaine speeded up wound closure and histological regeneration partially by attenuating oxidative stress and protecting cells from oxidative damage.

**FIGURE 7:**
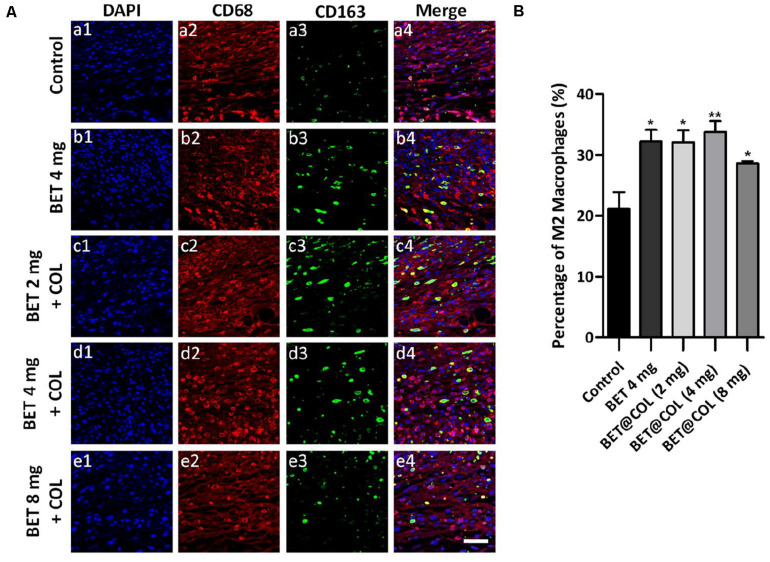
Betaine promoted M2 macrophages polarization in wounds on day seven. **(A)** Fluorescence photomicrographs of M2 macrophages of the control, free betaine solution treated and BET@COL treated wounds. Scale bar is 50 μm. **(B)** Quantitative result of immunofluorescence staining, *n* > 5. BET, betaine; COL, collagen. Statistical differences were performed using ANOVA. **p < 0.01, *p < 0.05, compared to control group.

The inflammation phase is a critical part of the wound healing process, while the exaggerated and prolonged inflammatory response has been proposed to be pathogenic mechanism of chronic wounds. During the inflammation period, macrophages infiltration is an indispensable link, mainly consisting of M1 macrophages (the pro-inflammatory phenotype) and M2 macrophages (the anti-inflammatory phenotype). The polarization toward M2 macrophages prompted the resolution of inflammation and benefited wound healing (Yin et al., 2013; He et al., 2017). As mentioned above, inflammation is closely coupled to oxidative stress. The two responses promote each other with complicated underlying mechanisms. We assumed that the alleviation of oxidative stress levels by betaine would reduce the inflammatory response in the wounds in the meantime. In order to verify our assumption, we evaluated the inflammation levels by calculating the percentage of M2 macrophages. CD68 was chosen as a marker of macrophages for all subtypes and CD163 for M2 macrophages (Labonte et al., 2014). As illustrated in Figure 7A, the control group had a low level of CD163 expressing cells while the free betaine (4 mg) solution and BET@COL treated groups exhibited higher levels. Significantly increased levels of M2 macrophages by 1.59-fold in BET@COL (4 mg) (33.76 ± 1.82%, *p* < 0.01) and by 1.52-fold in free betaine solution (4 mg) group (32.20 ± 1.93%, *p* < 0.01) compared with the untreated group (21.17 ± 2.74%) were demonstrated in Figure 7B, indicating enhanced polarization of M2 macrophages and a restraint of inflammatory response by betaine application.

## Conclusion

A moist and bioactive microenvironment plays a critical role in determining wound healing fate. While zwitterionic betaine has demonstrated its superior water binding and antifouling properties, the less efforts and progress have been made to study its effect on dermal wound healing. In this work, we proposed to incorporate betaine into BET@COL collagen sponges to improve their water retention ability, multiple cell biocompatibility *in vitro*, and wound healing efficiency *in vivo*. First, betaine alone demonstrated its superior cell biocompatibility to reduce oxidative damage on NIH 3T3 fibroblasts with an improved survival rate when exposed to H_2_O_2_, and more specifically, to significantly prevent mitochondria damage caused by H_2_O_2_ stimulation in NIH 3T3 fibroblasts. Further, upon incorporation of betaine into collagen sponges and applying the resultant BET@COL collagen sponges to a full-thickness wound mice model, the sponges demonstrated their accelerated wound healing efficiency as evidenced by enhanced wound closure, accelerated granulation tissue formation, increased collagen deposition and improved new blood vessel formation. Such high wound healing efficiency is likely attributed to the betaine-induced high water retention, low oxidative stress and low inflammation. The results indicate that the betaine-based materials could hold promise for wound dressing applications.

## Data Availability Statement

The datasets generated for this study are available on request to the corresponding author.

## Ethics Statement

The animal study was reviewed and approved by the Ethics Committee of Wenzhou Medical University and followed the International Ethical guidelines and the National Institutes of Health Guide concerning the Care and Use of Laboratory Animals.

## Author Contributions

HH, JZ, and JW proposed and designed the project. AC, YA, WH, and TX synthesized and fabricated sponges. AC, YA, MY, and SL performed cell and tissue tests. AC, YA, SL, and XX performed mice model. All authors participated in result analysis and discussion and manuscript writing.

## Conflict of Interest

The authors declare that the research was conducted in the absence of any commercial or financial relationships that could be construed as a potential conflict of interest.
