# Supplementary material for: Highly Water-Preserving Zwitterionic Betaine-Incorporated Collagen Sponges With Anti-oxidation and Anti-inflammation for Wound Regeneration
Source: Front Cell Dev Biol. 2020 Jul 15;8:491. doi: 10.3389/fcell.2020.00491 (PMC7381158; doi:10.3389/fcell.2020.00491)
Supplement: Supplementary file 1 [file Image_1.pdf]

Supporting Information

Highly Water-Preserving Zwitterionic Betaine-incorporated Collagen Sponges with Anti-oxidation and Anti-inflammation for Wound Regeneration

Anqi Chen[1†], Ying An[1†], Wen Huang[1], Tengxiao Xuan[1], Qianwen Zhang[2,3], Mengqi Ye[2], Sha Luo[2], Xuan Xuan[3], Huacheng He[2*], Jie Zheng[4,5*], Jiang Wu[1*]

[1]School of Pharmaceutical Sciences
Key Laboratory of Biotechnology and Pharmaceutical Engineering
Wenzhou Medical University, Wenzhou, 325035, China

[2]College of Chemistry and Materials Engineering
Wenzhou University, Wenzhou, 325027, China

[3]Department of Dermatology
The First Affiliated Hospital of Wenzhou Medical University, Wenzhou, 25000, China

[4]Department of Chemical, Biomolecular, and Corrosion Engineering
The University of Akron, OH, 44325, USA

[5]Department of Polymer Engineering
The University of Akron, OH, 44325, USA

[#]These authors contributed equally to this work.

* Corresponding Author: hehc@wzu.edu.cn (HH); zhengj@uakron.edu (JZ); woody870402@hotmail.com (JW)

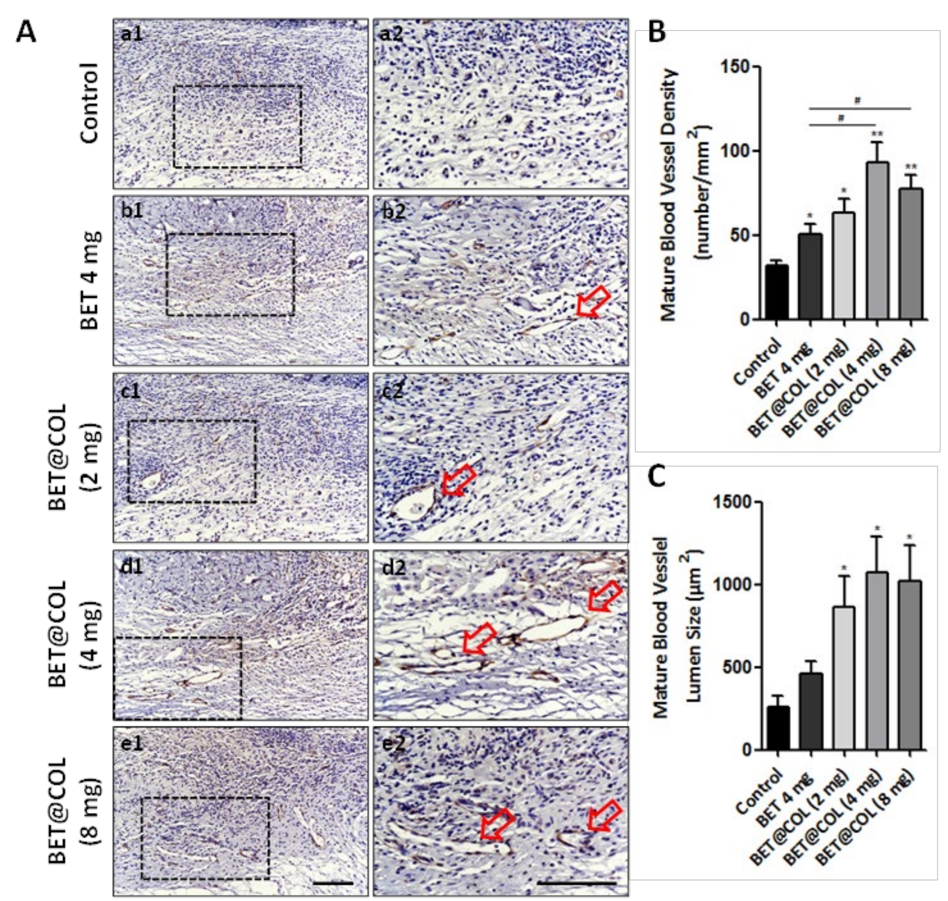

Figure S1. BET@COL wound dressing enhances vascular regeneration and maturation. (A) Representative images of immunohistochemical staining of α-SMA (α-smooth muscle actin) for the control group, free betaine solution (4 mg), and BET@COL (2 mg, 4 mg and 8 mg) groups on day seven. Red arrows indicate mature blood vessels with large lumen sizes. Scale bars = 100 μm. **(B)** Quantitative results of the mature blood vessel density (number/mm^2) for (A). n = 3. **(C)** Quantitative results of the mature blood vessel lumen size (μm^2) for (A). n > 3. BET: betaine. COL: collagen. Statistical differences were performed using ANOVA. ** $p < 0.01$, * $p < 0.05$, compared to control group, # $p < 0.05$.
